# Bibliometric visualization analysis of the application of artificial intelligence in gastrointestinal surgery recent 10 years

**DOI:** 10.1007/s13304-025-02465-x

**Published:** 2025-12-09

**Authors:** Xu Wang, Mengya Dong

**Affiliations:** 1 Department of Gastrointestinal Surgery, Huzhou Central Hospital, Huzhou, China; 2 Department of Emergency Medicine, Huzhou Central Hospital, Huzhou, China

**Keywords:** Gastrointestinal surgery, Artificial intelligence, Deep learning, Bibliometrics, CNN

## Abstract

To systematically analyze the current status and future trends of artificial intelligence (AI) in gastrointestinal surgery from July 2015 to July 2025 through bibliometrics. Based on the Web of Science Core Collection database, this study retrieved relevant literature from July 13, 2015 to July 13, 2025, and used CiteSpace and VOSviewer software to visualize 1697 articles by country/region, institution, author, journal, fund and keyword. The results show that the number of publications in this field peaked in 2024, with 378 papers and 30,555 cumulative citations. China emerges as the predominant contributor to research output, with 797 papers, 14,075 citations, and an average of 17.66 citations per paper. However, the United States exhibits a higher average citation count per paper, at 26.62, and 6523 total citations. Notably, Tian Jie, with 1921 citations, and Li Jing, with 31 publications, are prominent scholars from China in this domain. Keyword analysis reveals a transition from foundational AI algorithms to clinical precision applications. While China holds a leading position in terms of research output, there is a need for enhancement in research quality. The field is currently experiencing an upward trajectory, with future research likely to focus on advancements in AI-assisted individualized precision medicine.

## Core tip

Based on the Web of Science database, this paper conducts a bibliometric analysis of 1697 articles on the application of artificial intelligence (AI) in gastrointestinal surgery. The results show that the amount of literature in this field continues to grow, reaching a peak in 2024 (378 articles), with a total of 30,555 citations. China leads in the number of publications (797 articles) and total citations (14,075 times), but the United States has a higher average citation (26.62 times). The research topic has shifted from basic AI algorithms to clinical precision applications, forming a “technology-disease-treatment” trinity framework. In the future, AI-assisted personalized precision medicine will become a focus.

## Introduction

The history of gastrointestinal surgery can be traced back to ancient civilizations. The advent of modern physiology, anatomy, and aseptic techniques has significantly advanced surgical methodologies, particularly in colorectal surgery. In ancient times, the high bacterial load within the intestines often led to postoperative infections and increased mortality rates, as the concept of aseptic technique was not yet established [1]. In contemporary gastrointestinal surgery, the most transformative innovation is minimally invasive surgery, characterized by small incisions, rapid wound healing, reduced patient pain, fewer complications, and significantly shorter hospital stays [2]. Comparing to traditional open surgery, laparoscopic procedures for colorectal and gastric cancers are characterized by smaller incisions, which consequently reduce the risk of postoperative pain and infection. The long-term efficacy of both surgical approaches is comparable; however, laparoscopic surgery significantly decreases postoperative hospitalization duration and accelerates patient recovery [3, 4]. Concurrently, the utilization of robotic surgery in gastrointestinal procedures has increased in recent years, with numerous studies affirming its safety and feasibility. A prospective study conducted by Elisabeth Myrseth et al. demonstrated that robot-assisted rectal resection exhibits a lower conversion rate to laparotomy compared to traditional laparoscopic surgery. The findings of this study indicate that robotic surgery offers enhanced safety, stability, and surgical completion [5]. Alongside advancements in surgical techniques, significant progress has also been made in the diagnosis, adjuvant therapy, and perioperative management of gastrointestinal tumors. For instance, Enhanced Recovery After Surgery (ERAS) protocols have gained widespread acceptance as the preferred postoperative treatment strategy in colorectal surgery, showcasing distinct advantages in gastrointestinal surgical procedures [6].

The advancement of precision gastrointestinal surgery is presently hindered by several challenges. Preoperative endoscopic and imaging diagnostics are predominantly dependent on the clinician’s expertise, which introduces subjective biases and the potential for missed diagnoses. During surgery, the intricate anatomical structures and the demands of minimally invasive techniques impose significant challenges on the surgical team. Postoperatively, the accurate prediction of patient complications and recurrence risk necessitates personalized precision strategies, while the entire perioperative period demands a meticulously integrated and scientifically informed medical approach. Consequently, artificial intelligence technology is progressively emerging as a pivotal tool in overcoming these obstacles and facilitating intelligent diagnostic and therapeutic decision-making. For instance, AI-driven deep learning algorithms have markedly enhanced the diagnostic accuracy of gastrointestinal endoscopic lesions, thereby improving diagnostic efficiency. AI assistance also demonstrates superior adaptability and precision when addressing the complexities inherent in gastrointestinal endoscopic imagery [7, 8]. Furthermore, AI has been shown to increase the sensitivity and accuracy of endoscopic ultrasound (EUS) [9–11]. Beyond the diagnosis of gastrointestinal lesions, AI technology has been extended to surgical methodologies. For example, the da Vinci robotic surgery system employs AI to precisely compute and extract three-dimensional, high-definition images during surgical procedures, significantly enhancing surgical tolerance, safety, and precision [12]. The application of AI across various subfields of gastrointestinal surgery, such as disease diagnosis, surgical assistance, and risk prediction, presents promising prospects [13]. The number of related studies in this field is endless, but overall, the research results are relatively scattered, and there is a lack of a method to comprehensively and quantitatively analyze and sort out the current status of research in this field, the evolution of hot topics, and future trends.

Bibliometrics is a scientific approach that characterizes publication trends and elucidates inter-publication relationships [14]. In the medical domain, bibliometrics has emerged as a crucial instrument for assessing research quality [15]. Given the present circumstances, there is an urgent need to employ bibliometric methods to systematically analyze the visual publication landscape concerning the application of artificial intelligence in gastrointestinal surgery. Therefore, this study employs bibliometric methods to map the global landscape, identify research hotspots, and predict future directions in AI applied to gastrointestinal surgery.

## Materials and methods

### Data sources

This study is based on the Web of Science (WoS) database platform, and the literature is included in the core collection database publications in WOS. Since the publications in this database are all SCIE documents, its quality is currently considered to be the highest database category in the medical field and is often taken as the default analysis research object in bibliometrics.

### Search strategy

**Fig. 1:**
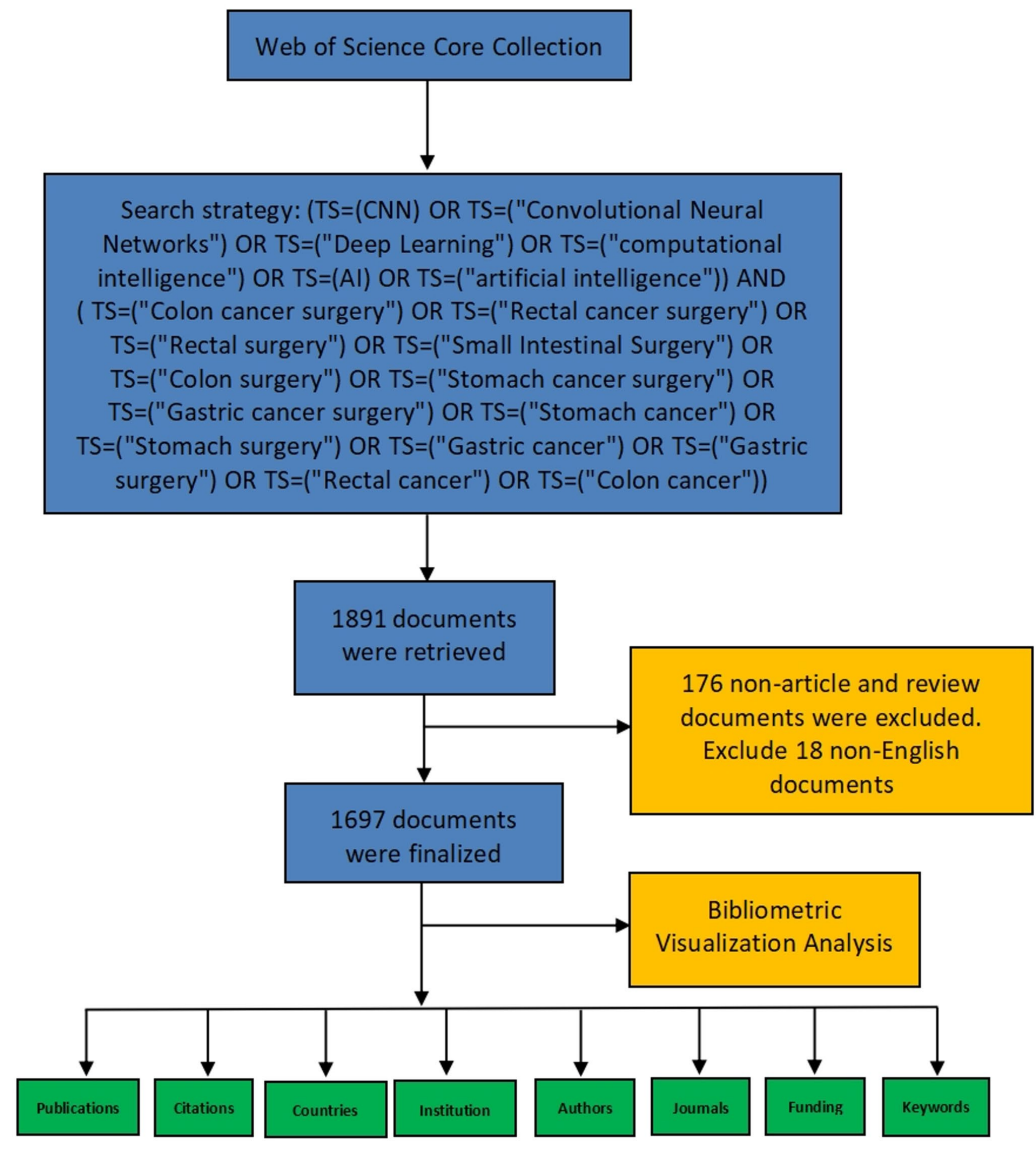
Flow chart of literature screening

The search methodology and strategy are depicted in Fig. 1. The search terms were designed to identify keywords associated with major surgeries pertinent to the subject areas of “artificial intelligence” and "gastrointestinal surgery." The search formula employed was as follows: (TS = (CNN) OR TS = ("Convolutional Neural Networks") OR TS = (“Deep Learning”) OR TS = (“computational intelligence”) OR TS = (AI) OR TS = (“artificial intelligence”)) AND (TS = ("Colon cancer surgery") OR TS = ("Rectal cancer surgery") OR TS = (“Rectal surgery”) OR TS = ("Small Intestinal Surgery") OR TS = (“Colon surgery”) OR TS = ("Stomach cancer surgery") OR TS = ("Gastric cancer surgery") OR TS = (“Stomach cancer”) OR TS = (“Stomach surgery”) OR TS = (“Gastric cancer”) OR TS = (“Gastric surgery”) OR TS = (“Rectal cancer”) OR TS = (“Colon cancer”)). The search period was defined from July 13, 2015, to July 13, 2025. To mitigate the impact of database updates on the search results, all searches were conducted within a single day, specifically on July 13, 2025. To ensure the novelty and relevance of the literature reviewed, the selection was restricted to monographs and review articles, while excluding conference abstracts, editorial papers, letters, and similar types. The language criterion was limited to English. In order to ensure the integrity of data preparation and prevent data collection errors, this study was conducted by two researchers to export and fully save the data and check each other, mainly analyzing eight aspects such as "number of publications", "number of citations", "country/region", “institution”, “author”, “journal”, “fund”, and “keywords”. If there are inconsistencies in the statistical data, they will be checked by a third person.

### Analysis software

This study primarily employs two software tools for statistical analysis. The first is CiteSpace, developed by Chaomei Chen and colleagues at Drexel University in the United States in 2005. CiteSpace is designed for the visual quantitative analysis of literature data [16]. It integrates visualization technology with traditional evidence-based medicine methodologies to examine networks of authors, institutions, and research hotspots within a specific field. This allows practitioners to discern the status of research collaborations, identify mainstream topics, and explore emerging directions in the field. The second software utilized in this study is VOSviewer, a bibliometric visualization tool created by Van Eck and Waltman at Leiden University in the Netherlands in 2010 [17, 18]. VOSviewer facilitates the visualization of network cooperation and the analysis of hotspot distribution, as well as temporal distribution analyses for authors, institutions, countries/regions, and keywords within a particular domain. Overall, CiteSpace is adept at illustrating the relevance and prospective development trends within a field, whereas VOSviewer is more focused on deconstructing the cluster distribution among nodes. This study uses the advantages of both software to better explore the hot spot distribution, cutting-edge directions, and cooperation trends in this field.

## Results

### Annual publication

**Fig. 2:**
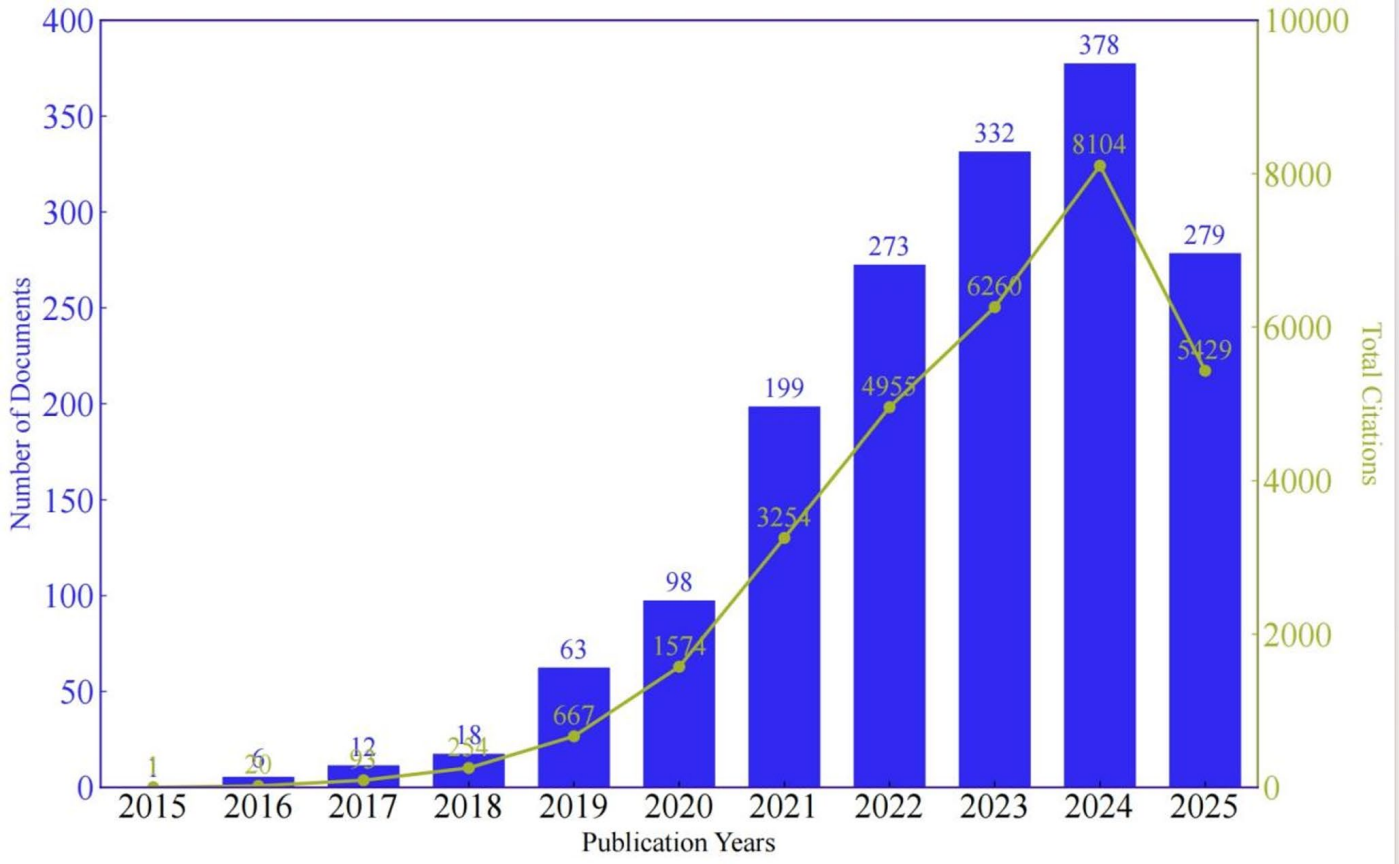
Number of publications and citations per year

Figure 2 presents a statistical analysis of the annual number of publications and total citations of the selected literature. The analysis indicates that between July 13, 2015, and July 13, 2025, a total of 1697 publications were identified from the Web of Science Core Collection database that met the inclusion criteria. These comprised 1400 monographs and 297 reviews, accumulating a total of 30,555 citations, with an average of 18.01 citations per article. The trend in publications has shown a consistent increase annually, with a notable surge between 2020 and 2021. The field experienced a peak in both the number of published articles and total citations in 2024, reaching 378 publications and 8104 citations, respectively. From January to July 2025, 279 articles have been included, and it is anticipated that the total number of publications in 2025 will surpass previous records.

### Country/region analysis

**Fig. 3:**
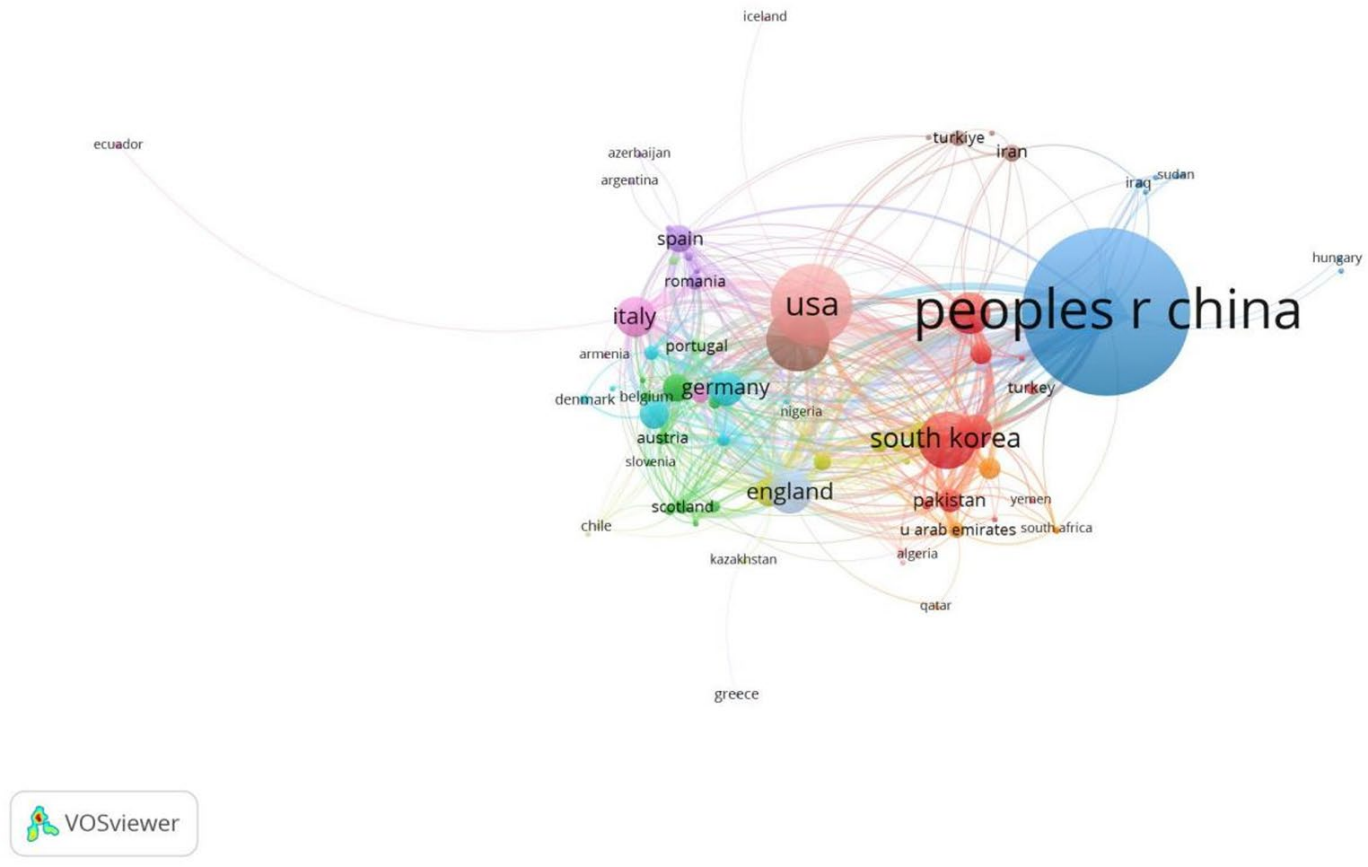
Country/region cooperation network

**Fig. 4:**
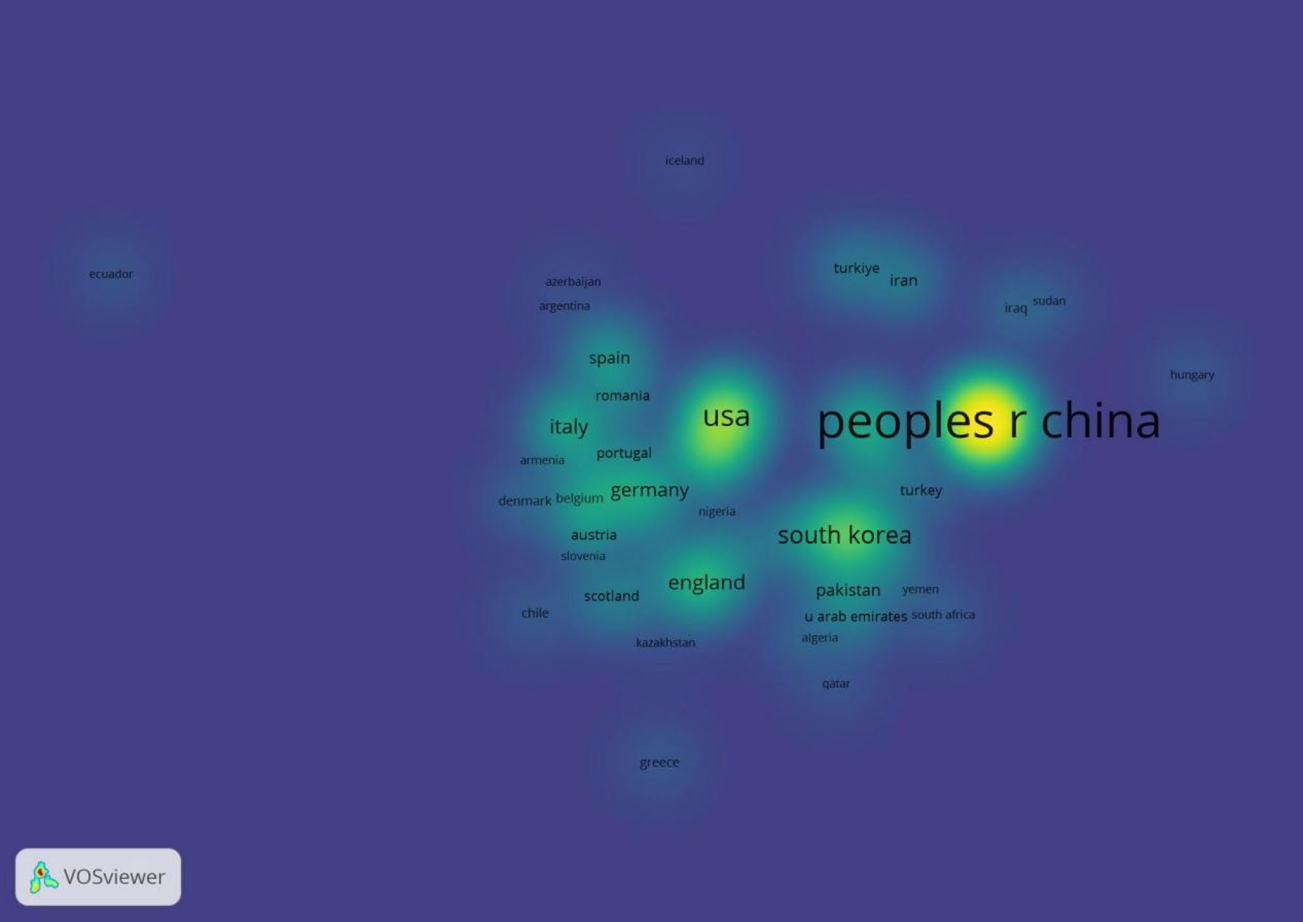
Country/region collaboration network heat map

This study conducted a cooperation network and hotspot analysis of countries/regions in this field (Figs. 3, 4). The results show that China occupies a leading position in this field, with the largest nodes and the most publications, followed by the United States, Japan, South Korea, England, etc. However, from the perspective of the frequency of cooperation links, it shows that the United States and South Korea have a higher frequency of cooperation with other countries, mainly Italy, Germany, Austria, Scotland, etc. From the cooperation hotspot map, there are three main hotspots centered on China, the United States, and South Korea. The hotspot distribution of Spain, Portugal, Italy, and Germany is centered on the United States, and the Chinese hotspot is relatively independent. The top five countries in terms of total citations of publications are China (14,075 times), the United States (6523 times), England (4875 times), Japan (4331 times), and Germany (2507 times) (Table 1). Among the top ten countries, 7 countries/regions are developing countries, while the remaining 3 countries/regions are developing countries. From the perspective of global distribution, there are 5 countries in Asia, 4 countries in Europe, and 1 country in the Americas.

**Table 1:** Top 10 countries by number of publications

Rank	Country/Region	Documents numbers	Total citations (without self-citations)	Average citation	H-index
1	China	797	14,075 (12,454)	17.66	54
2	USA	245	6523 (6392)	26.62	38
3	Japan	158	4331 (4087)	27.41	28
4	South korea	136	2139 (2031)	15.73	23
5	England	87	4875 (4809)	56.03	27
6	India	82	1230 (1260)	15	16
7	Italy	77	1484 (1459)	19.27	23
8	Germany	58	2507 (2453)	43.22	23
9	Saudi arabia	58	925 (872)	15.95	13
10	Netherlands	45	2028 (2013)	45.07	17

### Institution analysis

**Fig. 5:**
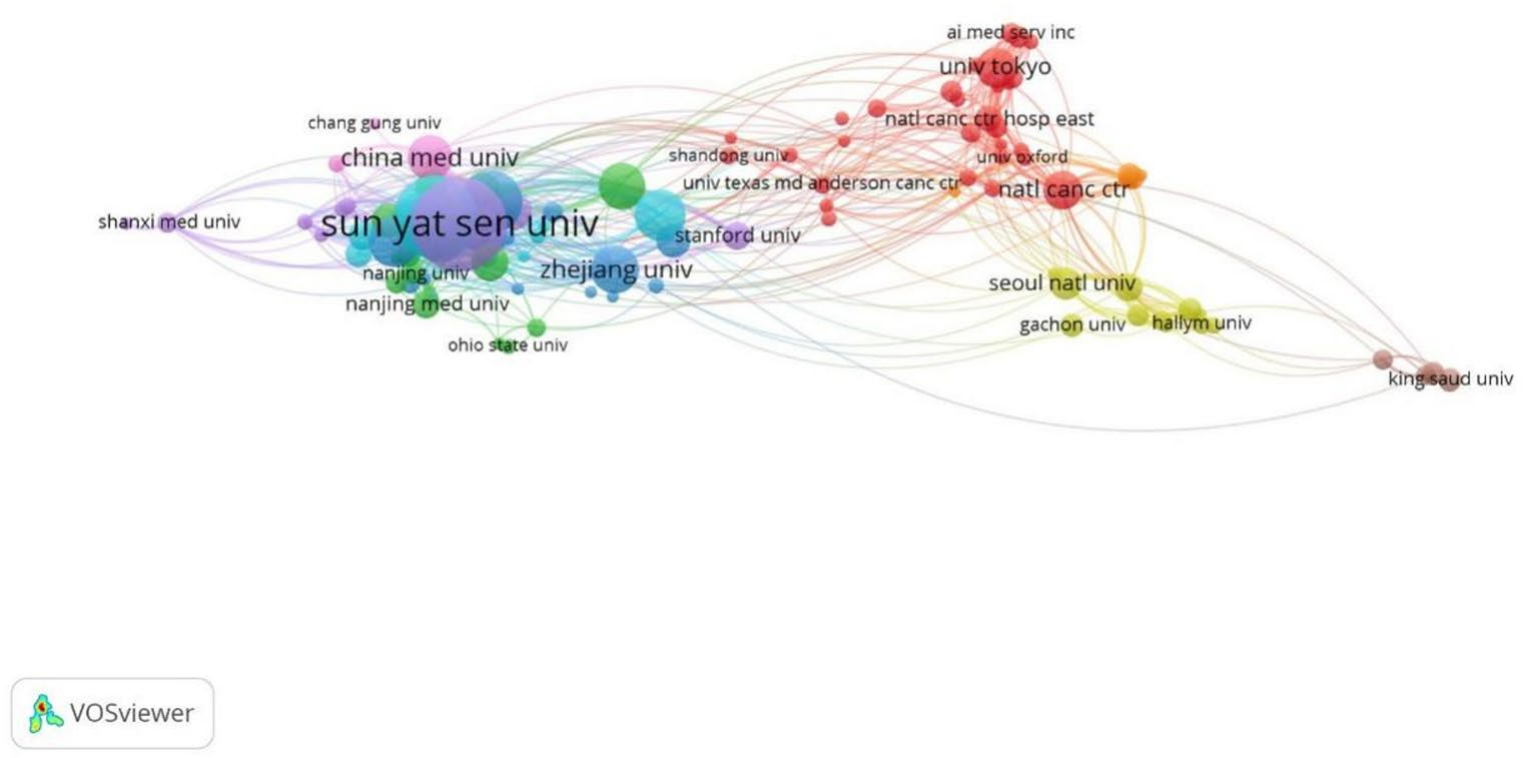
Institution cooperation network

**Fig. 6:**
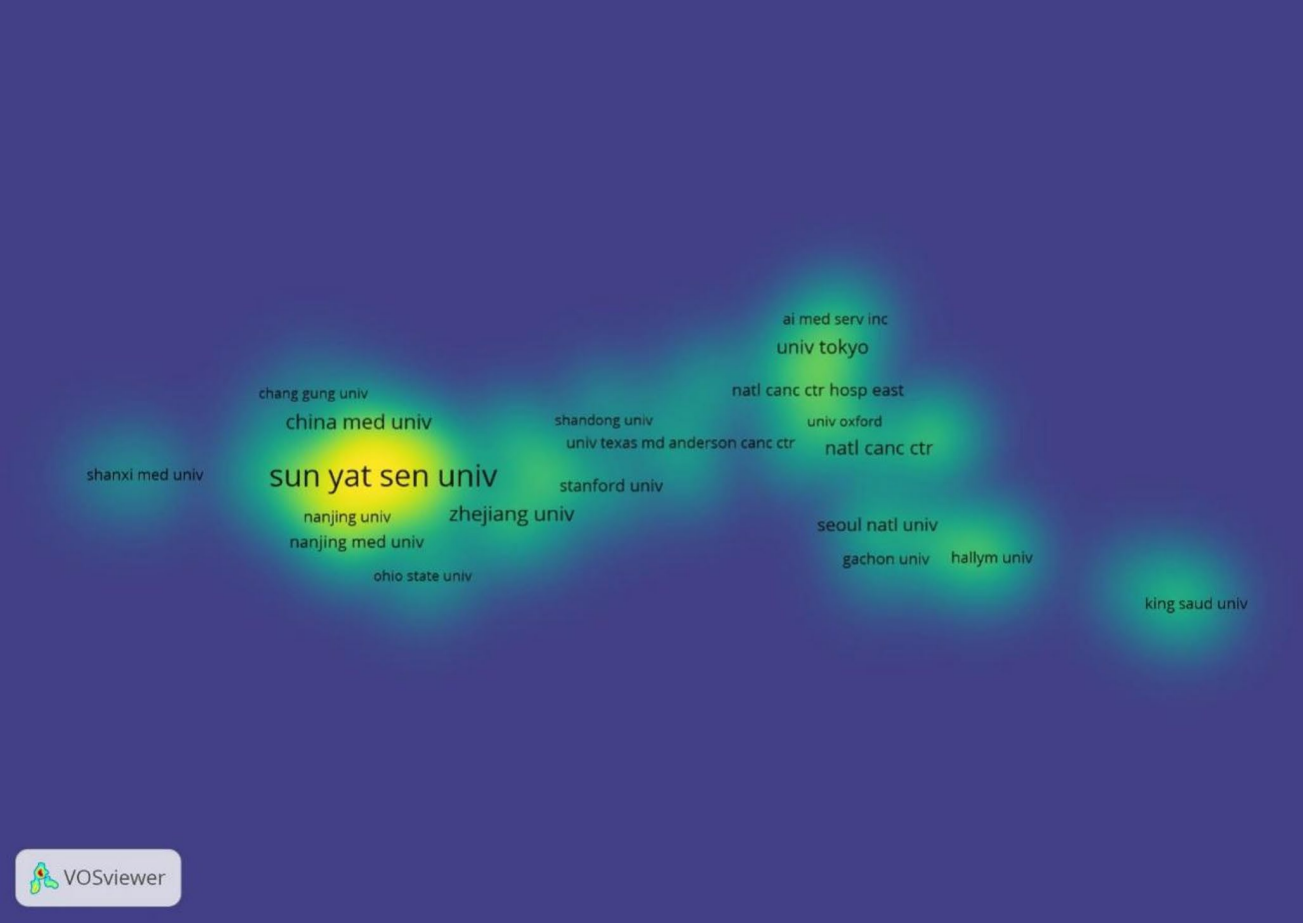
Institution collaboration network heat map

This study conducted a cooperation network and hotspot analysis of the institutions in this field (Figs. 5, 6). The cooperation network diagram shows that Sun Yat Sen University occupies a leading position in this field, with the largest node, and has close cooperation with Nanjing University, Nanjing Medical University, Zhejiang University and Stanford University. From the cooperation hotspot diagram, Chinese institutions, mainly Sun Yat Sen University and Nanjing University, show the main hotspot distribution. The top ten institutions in terms of the number of publications are Chinese Academy of Sciences (82 articles), Sun Yat Sen University (72 articles), Southern Medical University China (54 articles), Shanghai Jiao Tong University (46 articles), and University Of Chinese Academy of Sciences Cas (44 articles). The top five institutions in terms of total citations are Chinese Academy of Sciences (2895 times), Beihang University (2193 times), Sun Yat Sen University (2186 times), University Of Chinese Academy of Sciences Cas (2060 times), and National Cancer Center Japan (1683 times) (Table 2). Among the top ten institutions, 9 are from China and 1 is from Japan.

**Table 2:** Top 10 institutions by number of publications

Rank	Institution	Documents numbers	Country	Total citations (without self-citations)	Average citaiton	H-index
1	Chinese Academy of Sciences	82	China	2895 (2802)	35.3	24
2	Sun Yat Sen University	72	China	2186 (2131)	30.36	21
3	Southern Medical University China	54	China	1439 (1400)	26.65	17
4	Shanghai Jiao Tong University	46	China	647 (638)	14.07	14
5	University Of Chinese Academy of Sciences Cas	44	China	2060 (2008)	46.82	21
6	Beihang University	38	China	2193 (2150)	57.71	20
7	Fudan University	38	China	879 (864)	23.13	14
8	Sichuan University	36	China	565 (557)	15.69	12
9	Chinese Academy of Medical Sciences Peking Union Medical College	35	China	916 (909)	26.17	12
10	National Cancer Center Japan	35	Japan	1683 (1664)	48.09	17

### Author analysis

**Fig. 7:**
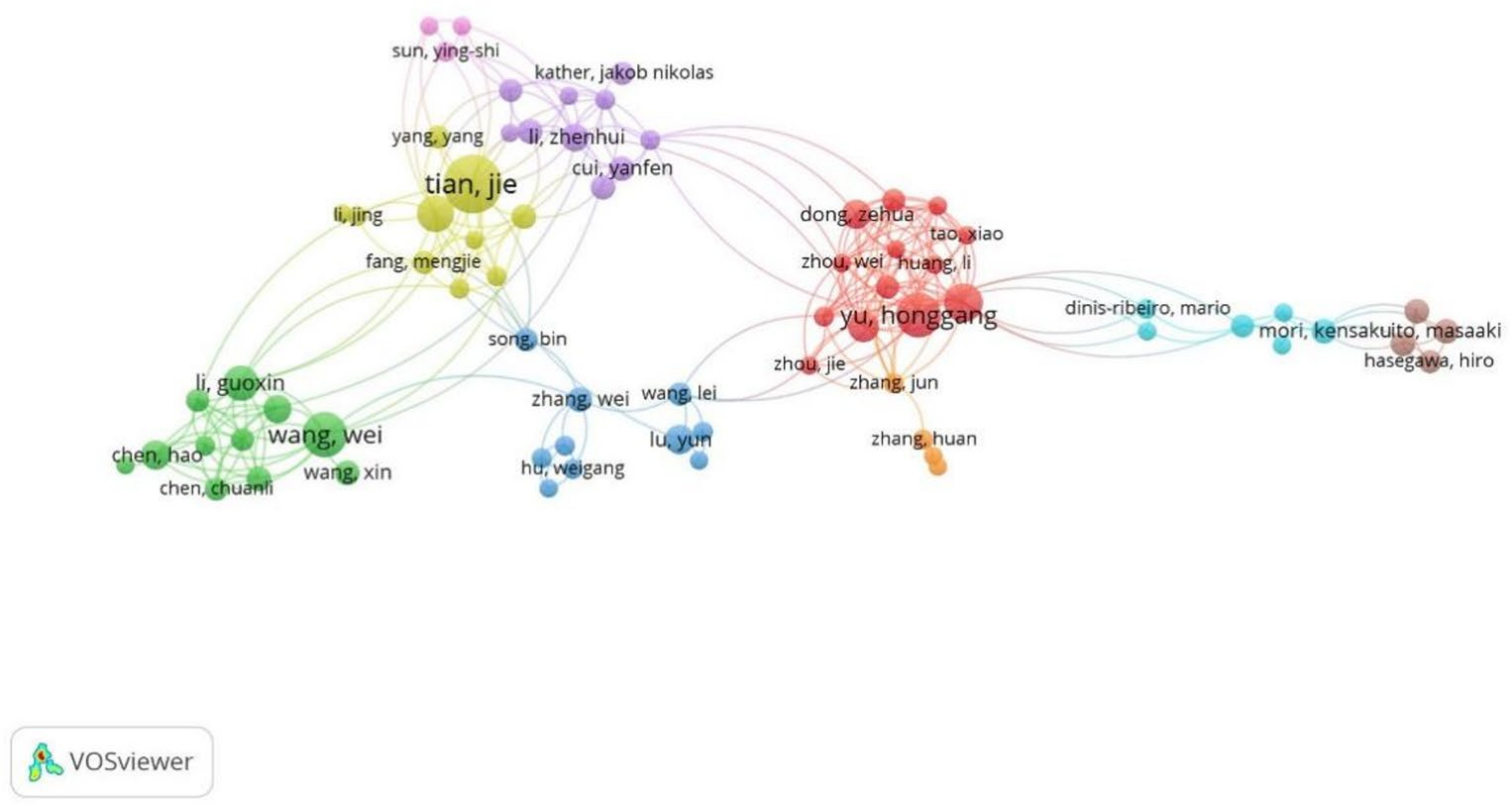
Author cooperation network

**Fig. 8:**
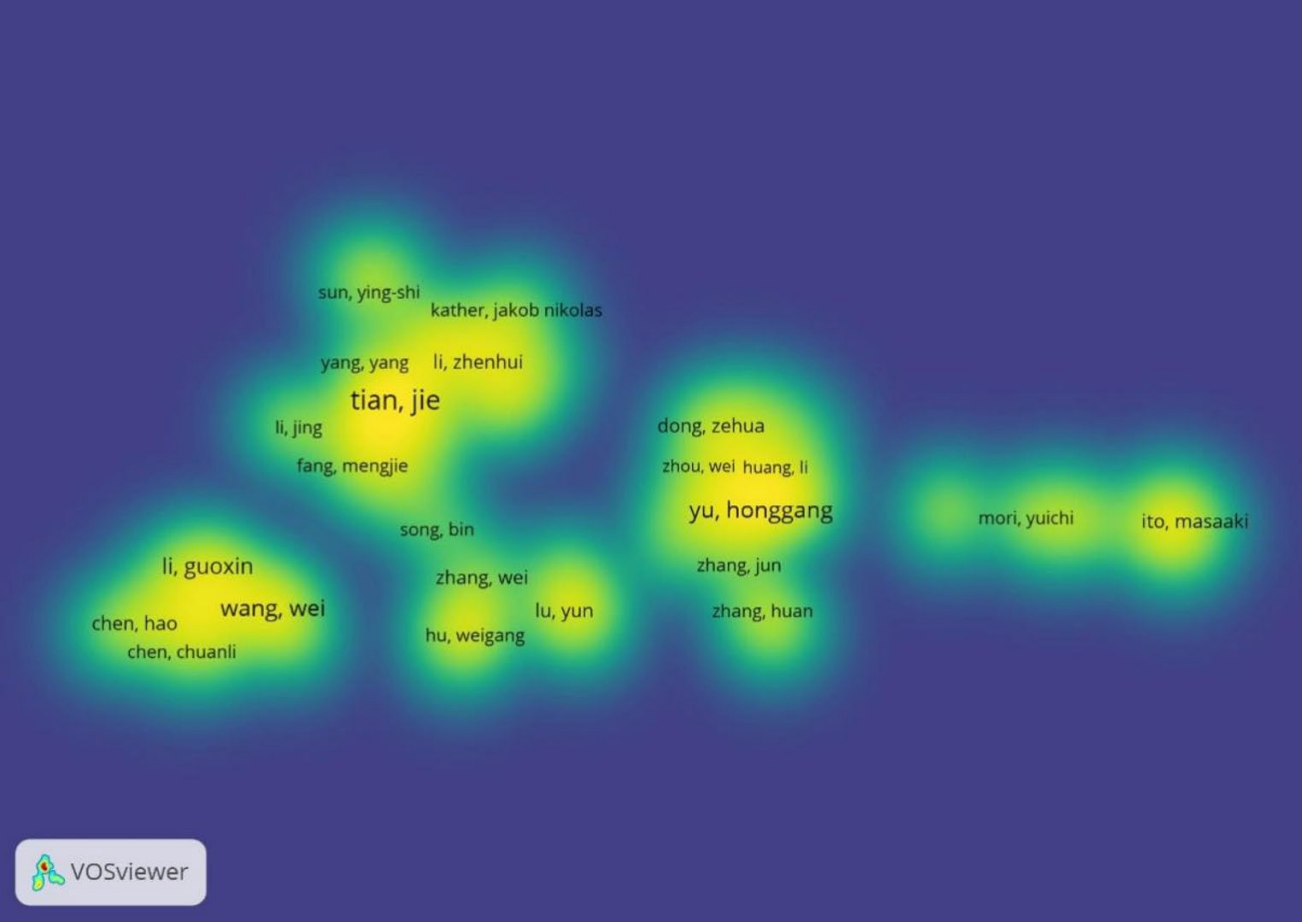
Institution collaboration network heat map

This study conducted a collaboration network and hotspot analysis on the authors in this field (Figs. 7, 8). The collaboration network diagram shows that Li Jing, Tian Jie, and Honggang Yu have the largest nodes in this field and the highest centrality status. Different colors also show the characteristics of cluster analysis of different authors, such as Li Jing and Tian Jie frequently collaborate, and Honggang Yu and Huang Li frequently collaborate. From the collaboration hotspot diagram, there are three main hotspot distributions with Tian Jie, Honggang Yu, Wang Wei and other authors as the main centers. The top three authors in terms of the number of publications are Li Jing (31 articles), Tian Jie (28 articles), and Zhenyu Liu (25 articles). The top three authors in terms of total citations are Tian Jie (1921 times), Zhenyu Liu (1458 times), and Li Jing (914 times) (Table 3). Among the top ten authors, all are from China.

**Table 3:** Top 10 authors by number of publications

Rank	Authors	Documents numbers	Affiliated institution, country	Total citations (without self-citations)	Average Citations	H-index
1	Li Jing	31	Department of Radiology, The First Affiliated Hospital of Zhengzhou University, Zhengzhou, Henan, China	914 (907)	29.48	10
2	Tian Jie	28	CAS Key Laboratory of Molecular Imaging, Institute of Automation, Chinese Academy of Sciences, Beijing, China	1921 (1877)	68.61	17
3	Zhenyu Liu	25	CAS Key Laboratory of Molecular Imaging, Institute of Automation, Beijing, China	1458 (1439)	58.32	11
4	Yong Liu	24	School of Computer Science and Technology, Heilongjiang University, Harbin, China	433 (432)	18.04	10
5	Xin Wang	23	Department of Biomedical Sciences, City University of Hong Kong, Hong KongSAR, China. 2. Shenzhen Research Institute, City University of Hong Kong, Shenzhen, China	746 (734)	32.43	13
6	Yang Li	22	Department of Pancreatic and Gastric Surgery, National Cancer Center/National Clinical Research Center for Cancer/Cancer Hospital, Beijing, China	467 (464)	21.23	8
7	Lu Wang	22	Chongqing Key Laboratory of Intelligent Oncology for Breast Cancer, Chongqing University Cancer Hospitaland School of Medicine, Chongqing University, Chongqing, China	530 (525)	24.09	11
8	Yan Wang	22	Shanghai Key Laboratory ofMultidimensional Information Processing,East China Normal University, Shanghai,China. 2. Engineering Center of SHMEC for SpaceInformation and GNSS, Shanghai, China	252 (250)	11.45	10
9	Honggang Yu	22	Key Laboratory of Hubei Province for Digestive System Disease and HubeiProvincial Clinical Research Center for Digestive Disease Minimally Invasive Incision, Renmin Hospital of WuhanUniversity, Wuhan, China	667 (628)	30.32	12
10	Xi Wang	21	1. Department of Radiation Oncology, Stanford University School of Medicine, Stanford, CA, USA 2. Department of Computer Science and Engineering, The Chinese University of Hong Kong, Hong Kong, China	270 (269)	12.86	8

### Journal and funding analysis

Table 4 shows the ranking of the top 10 journals in AI in the field of gastrointestinal surgery. The top three journals are Frontiers in Oncology (82 articles), Cancers and Diagnostics (53 articles each). However, in terms of total citations, Scientific Reports has the highest total citations (1206 times), and even after excluding self-citations, it still reaches 1198 times, with an average of 26.8 citations per article, and a high overall influence. In addition, Gastric Cancer (Q1, IF = 5.1) has an average of 38.54 citations per article, ranking first in academic influence per article. There are 6 journals in Q1 and 4 journals in Q2. Table 5 shows the names of the main funding funds in this field and the distribution of countries. Among the countries sponsored by the funds, China is the leading country (4 items), among which the National Natural Science Foundation of China (NSFC) leads the way with 342 articles (20.15%). The U.S. Department of Health and Human Services (52 papers) and NIH (51 papers) ranked second and third respectively, followed by Japan and South Korea. The top four funds funded more than 47 papers per institution, and the fifth and subsequent funds funded less than 44 papers, showing that fund sponsorship showed a head-concentrated effect.

**Table 4:** Top 10 journals by number of publications

Rank	Journal	Documents numbers	2024–2025 IF, JCR	Total citations (without self-citations)	Average Citations	H-index
1	FRONTIERS IN ONCOLOGY	82	3.3, Q2	671 (653)	8.18	15
2	CANCERS	53	4.4, Q2	821 (808)	15.49	16
3	DIAGNOSTICS	53	3.3, Q1	646 (630)	12.19	14
4	SCIENTIFIC REPORTS	45	3.9, Q1	1206 (1198)	26.8	15
5	IEEE ACCESS	33	3.6, Q2	454 (445)	13.76	11
6	SURGICAL ENDOSCOPY AND OTHER INTERVENTIONAL TECHNIQUES	29	2.7, Q1	334 (331)	11.52	11
7	JOURNAL OF GASTROINTESTINAL SURGERY	28	2.4, Q1	41 (41)	1.46	4
8	BIOMEDICAL SIGNAL PROCESSING AND CONTROL	26	4.9, Q2	219 (216)	8.42	9
9	GASTRIC CANCER	24	5.1, Q1	925 (922)	38.54	10
10	SURGERY	24	2.7, Q1	17 (17)	0.71	2

**Table 5:** The top 10 fundings by number

Rank	Documents numbers, percentage	Funding name	Country
1	342, 20.153%	National Natural Science Foundation of China Nsfc	China
2	52, 3.064%	United States Department of Health Human Services	USA
3	51, 3.005%	National Institutes of Health Nih Usa	USA
4	47, 2.770%	National Key R D Program of China	China
5	44, 2.593%	National Research Foundation of Korea	South korea
6	42, 2.475%	Japan Society for The Promotion of Science	Japan
7	42, 2.475%	Ministry Of Education Culture Sports Science and Technology Japan Mext	Japan
8	37, 2.18%	Grants In Aid for Scientific Research Kakenhi	Japan
9	31, 1.827%	Beijing Natural Science Foundation	China
10	31, 1.827%	National Key Research Development Program of China	China

### Keyword hotspot analysis

**Fig. 9:**
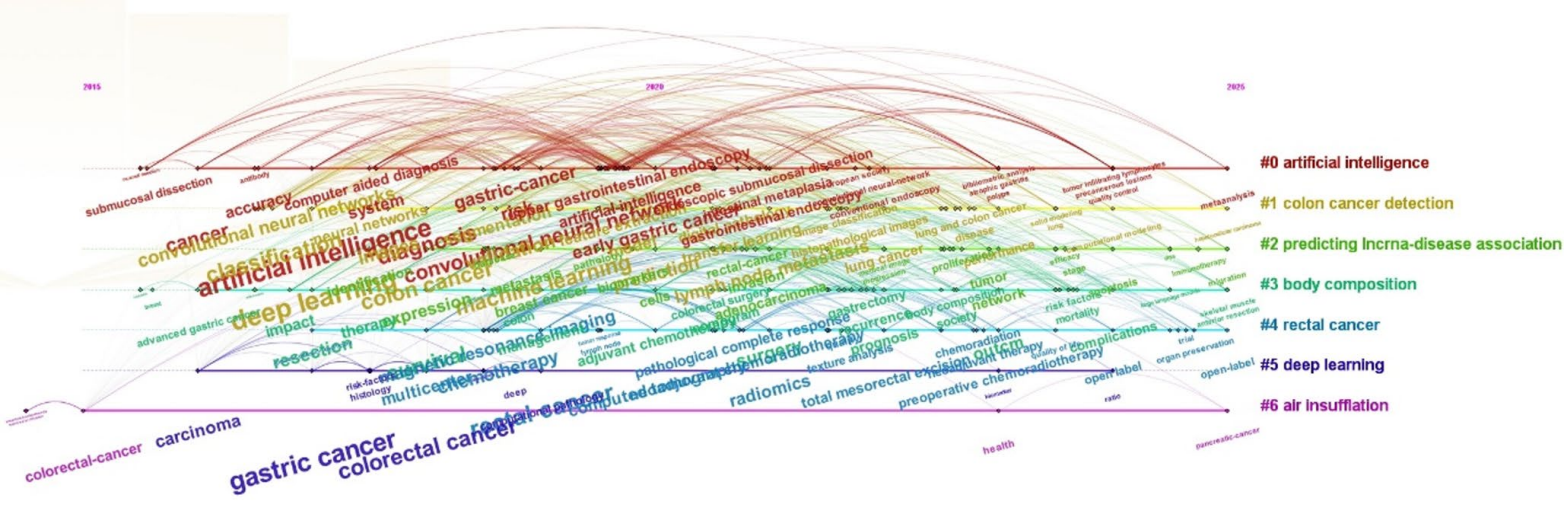
Keyword hotspot time evolution diagram

The keywords exhibiting the highest frequency of co-occurrence within this domain include “deep learning” (518 instances), “artificial intelligence” (462 instances), “gastric cancer” (412 instances), “rectal cancer” (208 instances), “colorectal cancer” (176 instances), “diagnosis” (167 instances), “classification” (162 instances), “colon cancer” (161 instances), “machine learning” (160 instances), and “survival” (133 instances). These keywords elucidate the central thematic focus of artificial intelligence applications in gastrointestinal surgery. Figure 9 illustrates the temporal progression of keywords in this area of study. The research trajectory on this subject can be broadly categorized into two phases: 2015–2020 and 2020–2025. During the initial phase, the keywords "gastric cancer," "computer-aided diagnosis," and “resection” emerged, primarily concentrating on enhancing the diagnostic efficacy of AI-assisted imaging for gastrointestinal surgical tumors and the development of AI-based medical algorithm models. In the second stage, “risk factor” and “trial” gradually appeared, indicating that AI-assisted gastrointestinal surgical disease diagnosis in this field has gradually matured, and AI has begun to be used to assist in patient prognosis prediction trials and risk factor analysis, gradually using AI to transition to the stage of individual precision treatment exploration.

**Fig. 10:**
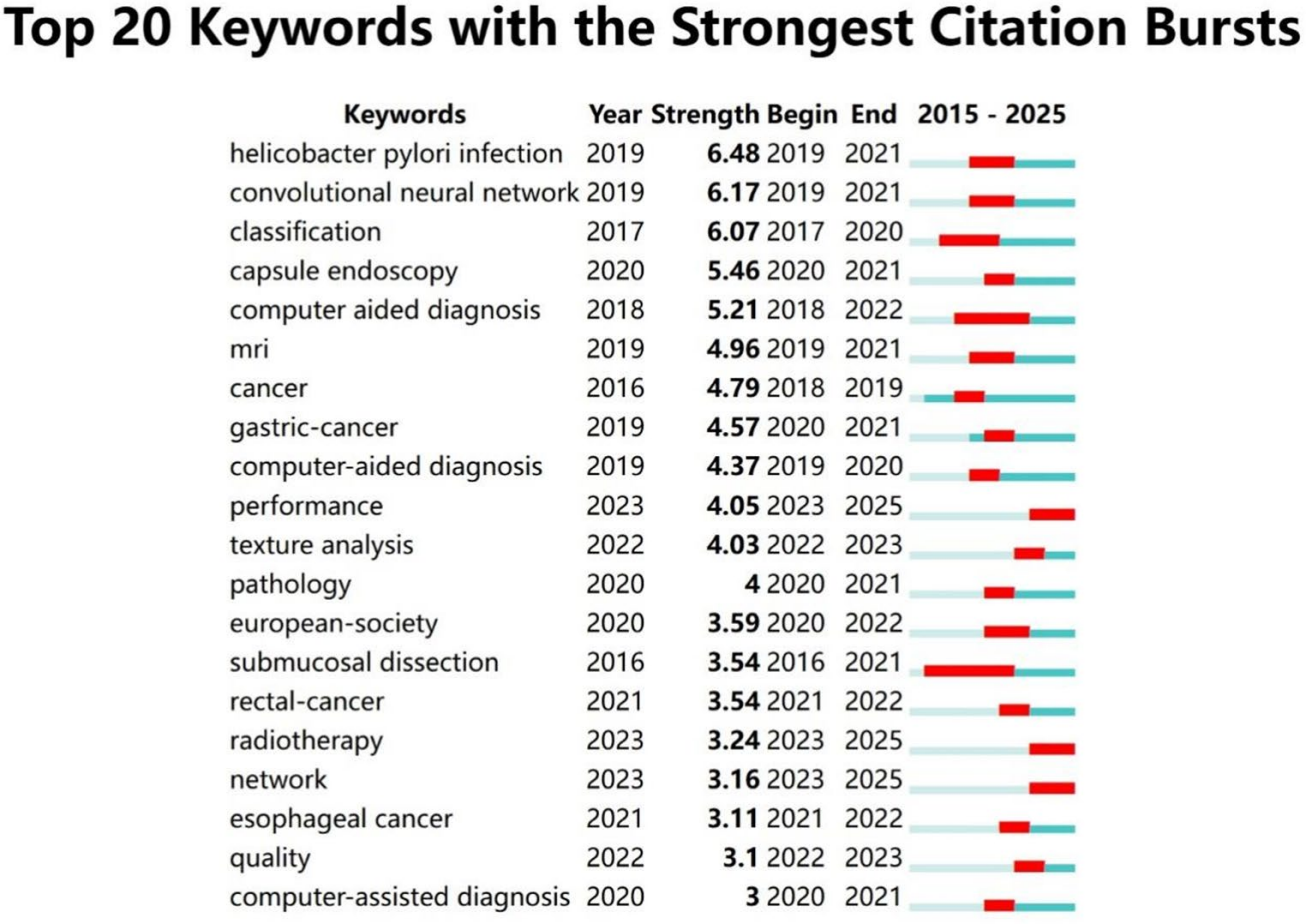
Keyword burst map

**Fig. 11:**
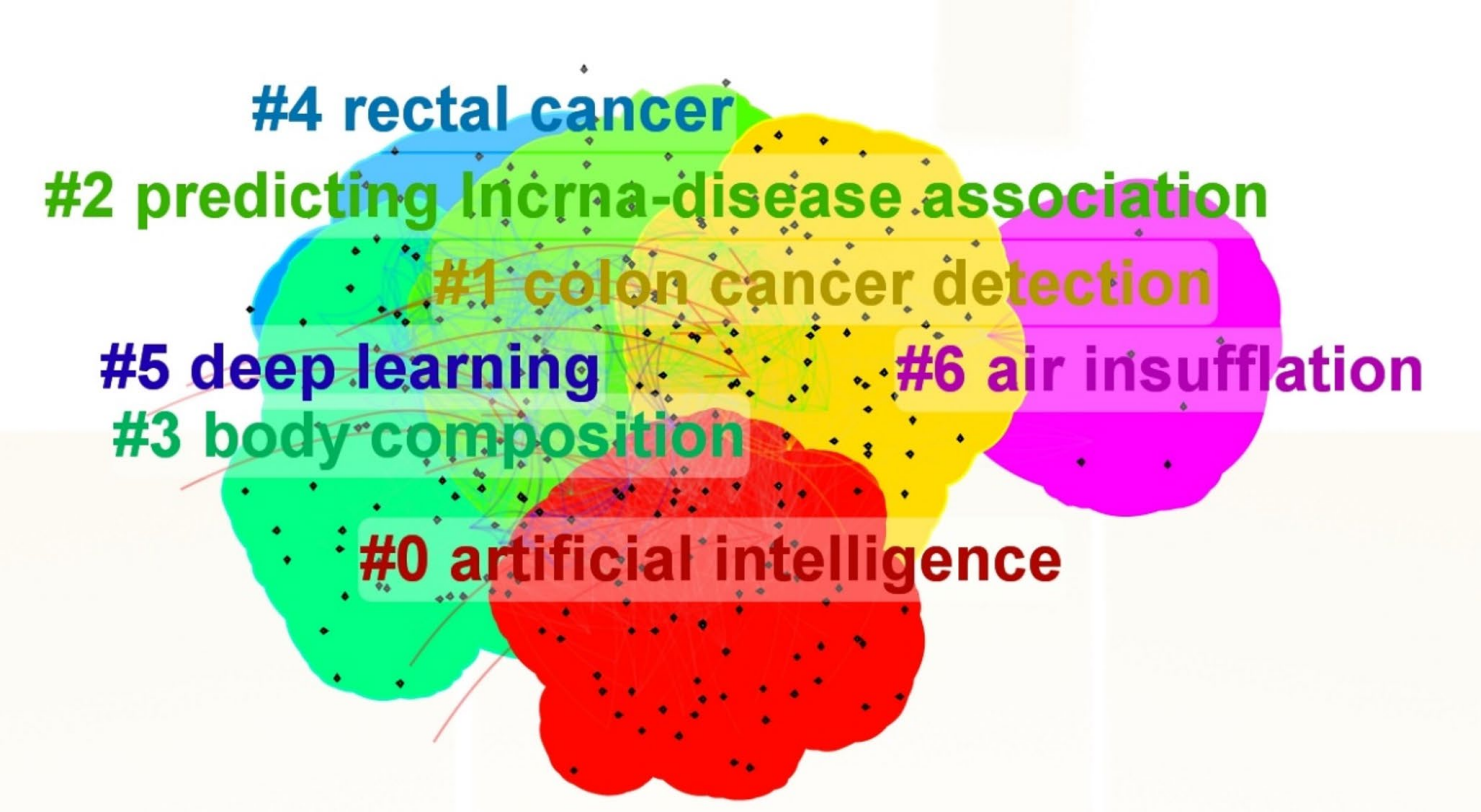
Keyword clustering diagram

Figure 10 illustrates the 20 most prominent keywords in the domain of medical artificial intelligence from 2015 to 2025. In 2019, "Helicobacter pylori infection" (6.48) and "convolutional neural network" (6.17) exhibited the highest levels of prominence. Between 2017 and 2023, keywords related to diagnostic technologies, such as “capsule endoscopy” and "computer-aided diagnosis," remained prevalent. Post-2023, new keywords, including “performance” and "radiotherapy," began to emerge. The keyword prominence chart delineates the developmental trajectory of this field, transitioning from foundational technologies (e.g., classification algorithms, CNN) to clinical applications (e.g., cancer diagnosis and treatment, quality assessment), aligning with the progression depicted in the timeline chart. Figure 11 is a cluster analysis of keywords. The clustered topics are closely related. AI technology (#0 Artificial Intelligence, #5 Deep Learning) drives precision cancer diagnosis and treatment (#4 Colorectal Cancer) and operation optimization (#6 Aeration), while extending to the molecular level (#2 Long-chain RNA disease prediction) and body evaluation (#3 Body composition), presenting us with the current status of scientific research of the "technology-disease-therapy" trinity.

## Discussion

### Global distribution and research quality

In the global landscape of this field, Chinese institutions dominate, accounting for 90% of the high-yield institutions, with national funding exceeding 20%. This scenario underscores China’s rapid advancement and dominant position, which are closely linked to substantial governmental support for the integration of artificial intelligence and medical care. The United States, which ranks second in terms of publication volume (245), demonstrates a higher average citation count per article at 26.62, compared to China’s 17.66. This disparity suggests a greater emphasis on research quality in the United States. However, it may also reflect the challenges faced by China due to its relatively recent entry into this field, which has yet to establish a comprehensive theoretical framework or achieve extensive international application. Consequently, China should enhance international technical collaboration and exchange to further elevate the quality of its research. The prominence of certain keywords highlights the significance of minimally invasive procedures, such as laparoscopic and robotic surgeries, within the realm of AI-assisted gastrointestinal surgery. This encompasses advancements in image and operation optimization. The temporal evolution diagram further illustrates a shift in focus within the field, from the exploration of AI algorithms and model development to the innovative clinical application of AI in "diagnosis-treatment-prognosis." Regarding established technologies, the emergence of keywords like capsule endoscopy (2020, burst intensity 5.46) and gas insufflation (cluster #6) underscores AI’s pivotal role in overcoming limitations in endoscopic technology [19] Furthermore, AI demonstrates its potential to support fundamental research across various disciplines. For instance, the molecular mechanism keyword cluster (#2 lncRNA disease prediction) indicates that researchers are leveraging AI as an auxiliary tool to facilitate the transition from macro-level surgical approaches to micro-level precision medicine in gastrointestinal surgery.

### Evolution of research themes

By analyzing the most cited publications and keywords, we can observe the evolution of research topics in this field. Our research findings indicate that the most frequently cited article in this domain is "Locality Sensitive Deep Learning for Detection and Classification of Nuclei in Routine Colon Cancer Histology Images," authored by Korsuk Sirinukunwattana et al. in 2016, with 844 citations. This study introduced a spatially constrained convolutional neural network (SC-CNN) for nucleus detection. The results demonstrated that the integration of the proposed SC-CNN with the NEP detection and classification method yields the highest average F1 score when compared to other existing methodologies [20]. This approach is anticipated to enhance the accuracy of slice image analysis in pathology, thereby significantly improving the precision of pathological diagnoses of colon cancer. The second most cited work is a 2019 publication by Jakob Nikolas Kather et al., titled "Deep learning can predict microsatellite instability directly from histology in gastrointestinal cancer," which has been cited 805 times. This research introduced an AI-based deep residual learning method for the direct prediction of microsatellite instability from standard H&E histological samples. Microsatellite instability plays a crucial role in determining the responsiveness of gastrointestinal cancer patients to immunotherapy, thereby eliminating the need for additional genetic or immunohistochemical testing. This study aims to enhance the accuracy of evaluations during the immunotherapy stage for gastrointestinal cancer patients [21]. The third most cited article, authored by Tian Jie et al. in 2019 and titled "The Applications of Radiomics in Precision Diagnosis and Treatment of Oncology: Opportunities and Challenges," has been cited 646 times. This work represents the most comprehensive evaluation to date of the primary methods for applying AI-assisted imaging in gastrointestinal tumors. It covers aspects such as data collection, tumor segmentation, feature extraction and modeling, as well as the rapidly evolving field of deep learning technology and its primary applications in tumor diagnosis, treatment, and prognosis evaluation [22]. This paper serves as the most authoritative reference standard for researchers in this domain and is considered an essential read within the industry. This study establishes a foundational reference for the clinical application of numerous subsequent AI algorithm models in gastrointestinal surgery [23–26]. The increasing application of artificial intelligence (AI) in this domain is evidenced by highly cited publications. In 2016, Sirinukunwattana et al. employed convolutional neural networks (CNN) for the analysis of colon cancer pathology slides, achieving automated diagnostic capabilities. By 2019, the scope of research had broadened to encompass Kather et al.'s work on predicting microsatellite instability through deep learning, as well as Tian Jie et al.'s comprehensive exposition on the application of radiomics in tumor diagnosis and treatment. This progression signifies the transformation of AI from a supplementary diagnostic tool to a pivotal enabler in guiding precise classification, treatment decisions, and prognostic assessments. The keyword timing and emergence analysis conducted in this study delineates the evolutionary trajectory of the field. During the initial phase (2015–2020), research predominantly concentrated on the development of foundational algorithms, including "convolutional neural networks (CNN)" and "deep learning," as well as their auxiliary diagnostic applications in “gastric cancer” and "colorectal cancer." In the subsequent period (2020–2025), the research focus transitioned toward treatment prognosis domains, encompassing areas such as "risk factors," "survival analysis," and "radiotherapy." This shift signifies a progression from algorithmic model development and disease-specific applications to addressing the more profound clinical needs of surgical optimization and personalized precision treatment.

### Clinical implications

Gastrointestinal diseases rank among the most prevalent conditions globally [27], and the evolution of gastrointestinal surgery is characterized by a rich historical context. Given the unique nature of these surgeries, facilitating a patient’s swift recovery post-operation can enhance nutritional intake and postoperative mobility, significantly mitigating the risk of complications such as postoperative infections and gastrointestinal dysfunction [28, 29]. Historically, challenges in the diagnosis of gastrointestinal tumors, surgical techniques, and perioperative management have posed significant obstacles. However, with the integration of AI, these bottlenecks are anticipated to be addressed, thereby establishing a robust foundation for the implementation of individualized, precision-based, comprehensive medical care.

This article acknowledges several limitations. Firstly, bibliometric analysis, being dependent on published works, is inherently time-sensitive and may overlook emerging topics anticipated in 2025. Additionally, recent research from 2025 may not yet have accrued a substantial number of citations, potentially leading to an underestimation of its influence and authority. More high-quality databases should be included in the future.

## Conclusion

The study indicates that over the past decade, China has led in terms of the scale of AI applications in gastrointestinal surgery, whereas the United States has produced higher-quality research. The evolving research hotspots illustrate a three-stage progression of AI integration with clinical practice, transitioning from the “technology layer” to the “disease layer” and finally to the "operation layer," alongside concurrent investigations into molecular mechanisms. The field is currently navigating the transformation toward optimizing AI-assisted minimally invasive surgery and advancing personalized precision diagnosis and treatment.

## Data Availability

No datasets were generated or analysed during the current study.
